# Effect of a Novel Postural Support Device Versus Conventional Swaddling on Postural Development in Preterm Neonates Over One Week: A Quasi-Experimental Trial

**DOI:** 10.7759/cureus.96680

**Published:** 2025-11-12

**Authors:** Dhwani D Chanpura, Nalina Gupta, Neha Mukkamala

**Affiliations:** 1 Pediatric Physiotherapy, College of Physiotherapy, Sumandeep Vidyapeeth Deemed to be University, Vadodara, IND; 2 Neurological Physiotherapy, Maharishi Markandeshwar College of Physiotherapy and Rehabilitation, Maharishi Markandeshwar University, Sadopur, IND; 3 Musculoskeletal Physiotherapy, College of Physiotherapy, Sumandeep Vidyapeeth Deemed to be University, Vadodara, IND

**Keywords:** infant positioning assessment tool, nicu, physiotherapy, postural development, preterm neonates, therapeutic positioning

## Abstract

Background: Preterm neonates are at high risk of postural asymmetries and developmental delays due to the abrupt transition from the protected intrauterine environment to the gravity-dominated neonatal intensive care unit (NICU). Therapeutic positioning is crucial for supporting early musculoskeletal and neuromotor development.

Objective: This study aimed to evaluate the effect of a novel postural support device, in combination with physiotherapy, on the postural development of preterm neonates in the NICU using the Infant Positioning Assessment Tool (IPAT).

Methods: A quasi-experimental trial with alternate group assignment was conducted with 146 preterm neonates (73 intervention and 73 control) admitted to a tertiary hospital NICU. Both groups received physiotherapy, but the intervention group was positioned using the newly developed postural support device, while controls received conventional swaddling. Postural alignment across multiple body segments was assessed weekly using IPAT.

Results: At baseline, no significant differences were observed between groups in demographic or clinical variables (p > 0.05). By the first week, the intervention group demonstrated significantly higher IPAT scores for shoulders (p < 0.001), hips/pelvis (p < 0.001), knees/ankles/feet (p < 0.001), and overall posture (p < 0.001) compared to controls. Improvements in head, neck, and hand positioning were also observed, with the intervention group showing early correction of baseline deficits.

Conclusion: The novel postural support device, when combined with physiotherapy, significantly enhanced postural symmetry and flexion in preterm neonates compared to conventional swaddling. These findings highlight the importance of structured, developmentally supportive positioning in optimizing early neuromotor outcomes for preterm infants in the NICU.

## Introduction

Preterm neonates, defined as infants born before the completion of 37 weeks of gestation, represent one of the most vulnerable populations in neonatal care [1]. The immaturity of their neurological and musculoskeletal systems places them at high risk of a wide range of developmental challenges [1]. Over the past few decades, advances in perinatal and neonatal medicine have led to remarkable improvements in the survival rates of preterm infants [2]. However, this increased survival has also been accompanied by a rise in short- and long-term morbidities, many of which stem from brain immaturity, structural anomalies, or injuries occurring during critical windows of neurodevelopment [2]. Conditions such as intraventricular hemorrhage, periventricular leukomalacia, and cerebral palsy are among the most common complications, often linked to long-lasting visual, auditory, cognitive, and motor impairments that can significantly influence a child’s overall quality of life [3].

One of the earliest and most pressing challenges for preterm neonates is adapting to life outside the intrauterine environment [4]. During the final trimester of pregnancy, the womb provides a warm, cushioned, and fluid-filled environment that naturally promotes a flexed posture, with the arms and legs positioned close to the body [4]. This fetal position not only conserves energy but also supports symmetrical musculoskeletal growth and neuromotor development [4]. With preterm birth, however, this protective environment is abruptly replaced by the uncontained and gravity-dominated surfaces of the neonatal intensive care unit (NICU) [4]. As a result, preterm neonates often struggle to maintain physiological flexion and instead adopt extended or asymmetrical postures [4]. These atypical positions contribute to poor joint alignment, abnormal movement patterns, muscle imbalance, and delays in achieving developmental milestones such as rolling, sitting, or independent head control [5]. Research has consistently shown that while only a small proportion of full-term infants exhibit motor impairments (2%-7%), the prevalence is dramatically higher in preterm populations, with estimates ranging from 54% to 64% [6]. Their spontaneous movements are often less fluent, less varied, and poorly coordinated, reflecting the challenges of an immature central nervous system confronted with abnormal postural stresses [6].

Postural stability is a cornerstone of motor development [6]. A stable and physiologically flexed posture supports effective motor planning, smoother coordination, and overall neurobehavioral organization [7]. However, preterm infants, with their low muscle tone and heightened sensitivity to gravity, frequently lie in hyperextended positions, which over time can reinforce asymmetry and hinder functional development [6,7]. This recognition has made therapeutic positioning a key component of neurodevelopmental care in the NICU. By recreating elements of the intrauterine environment, therapeutic positioning strategies provide containment, promote flexion, and help align the musculoskeletal system. These strategies are associated with multiple benefits, including improved tone regulation, enhanced comfort and self-soothing, better feeding performance, and stronger parent-infant bonding [8].

Traditional methods such as swaddling, nesting with rolled blankets, and diaper rolls are widely practiced and accessible [7]. However, they often lack the firmness and adaptability required to provide consistent support across different body regions, sometimes leading to leg abduction or trunk misalignment. Conversely, advanced positioning aids such as the Snuggle Up, Bendy Bumper, and Dandle Roo offer more structured containment but are costly and not always feasible in resource-limited NICU settings. This gap highlights the pressing need for an affordable, practical, and developmentally supportive postural device that can be used routinely in diverse healthcare contexts [8,9].

In response to this need, the present study was designed to develop and evaluate a novel postural support device intended to replicate intrauterine positioning while addressing the limitations of existing aids. The device was tested for its ability to promote symmetrical and flexed postures, thereby supporting early postural development. To objectively monitor outcomes, the Infant Positioning Assessment Tool (IPAT), a validated and standardized instrument, was employed to assess alignment and postural changes across multiple body segments during the NICU stay. This approach not only provides evidence on the effectiveness of the device but also adds to the growing body of knowledge on the role of positioning in the early development of preterm infants. Therefore, the objective of this study was to evaluate the effect of a novel postural support device combined with physiotherapy on postural alignment in preterm neonates, compared to conventional swaddling, during their NICU stay.

## Materials and methods

This study followed a non-randomized controlled clinical trial design and was conducted in the NICU of Dhiraj General Hospital, Vadodara, India. Written informed consent was obtained from the caregivers or guardians of all enrolled preterm neonates before the commencement of the study.

Preterm neonates were included based on specific criteria: gestational age between 30 weeks 0/7 days and 36 weeks 6/7 days, medical stability within the first 72 hours of life, and referral for physiotherapy. Exclusion criteria included the need for mechanical ventilation, continuous positive airway pressure (CPAP), or high-flow nasal cannula beyond 72 hours after birth, as well as any diagnosed neurological, musculoskeletal, genetic, chromosomal, or metabolic disorders.

Before initiating the intervention, each neonate underwent a routine physiotherapy assessment to determine clinical readiness for positioning and to ensure suitability for participation in the study.

**Figure 1:**
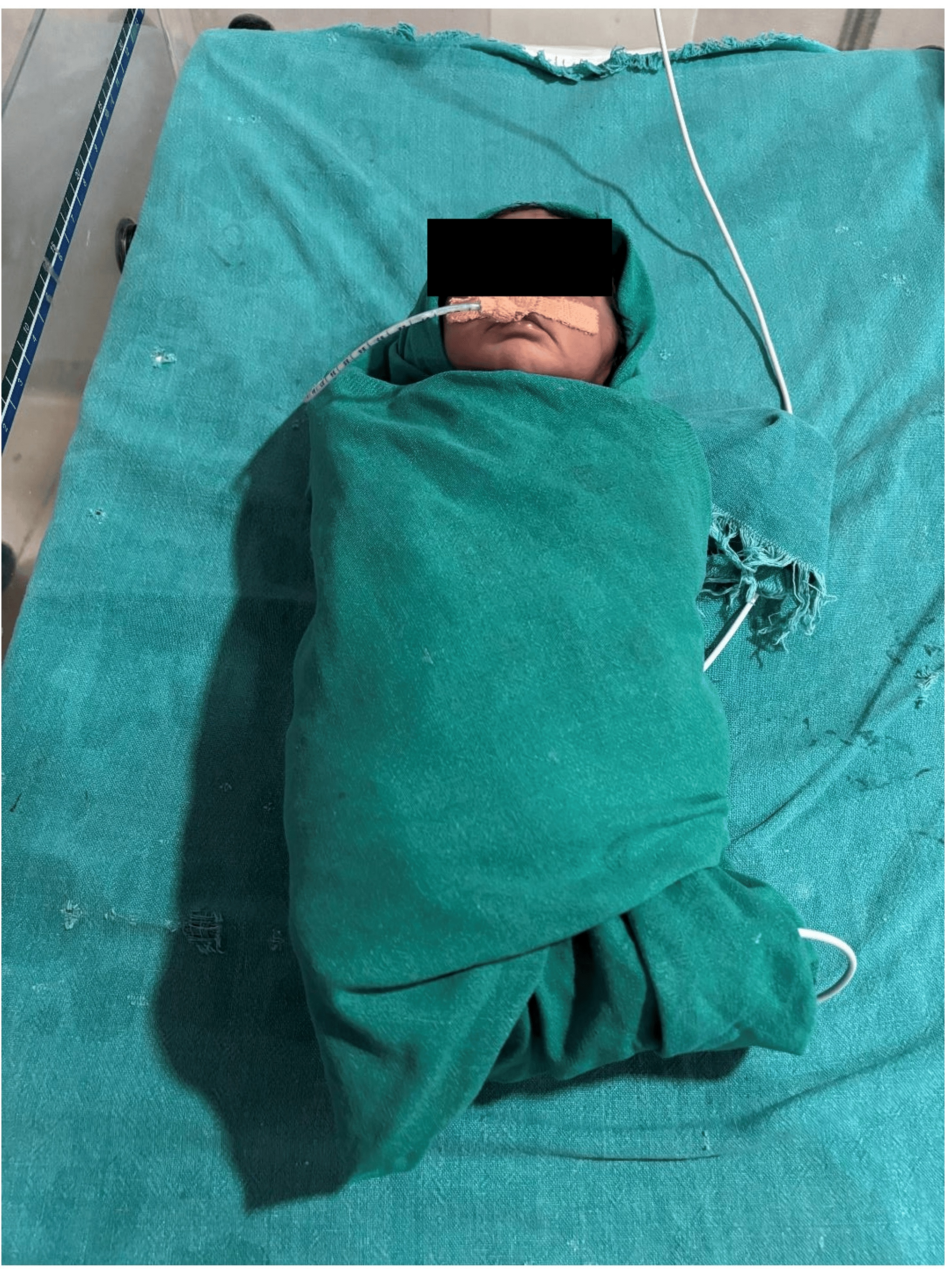
Conventional positioning

**Figure 2:**
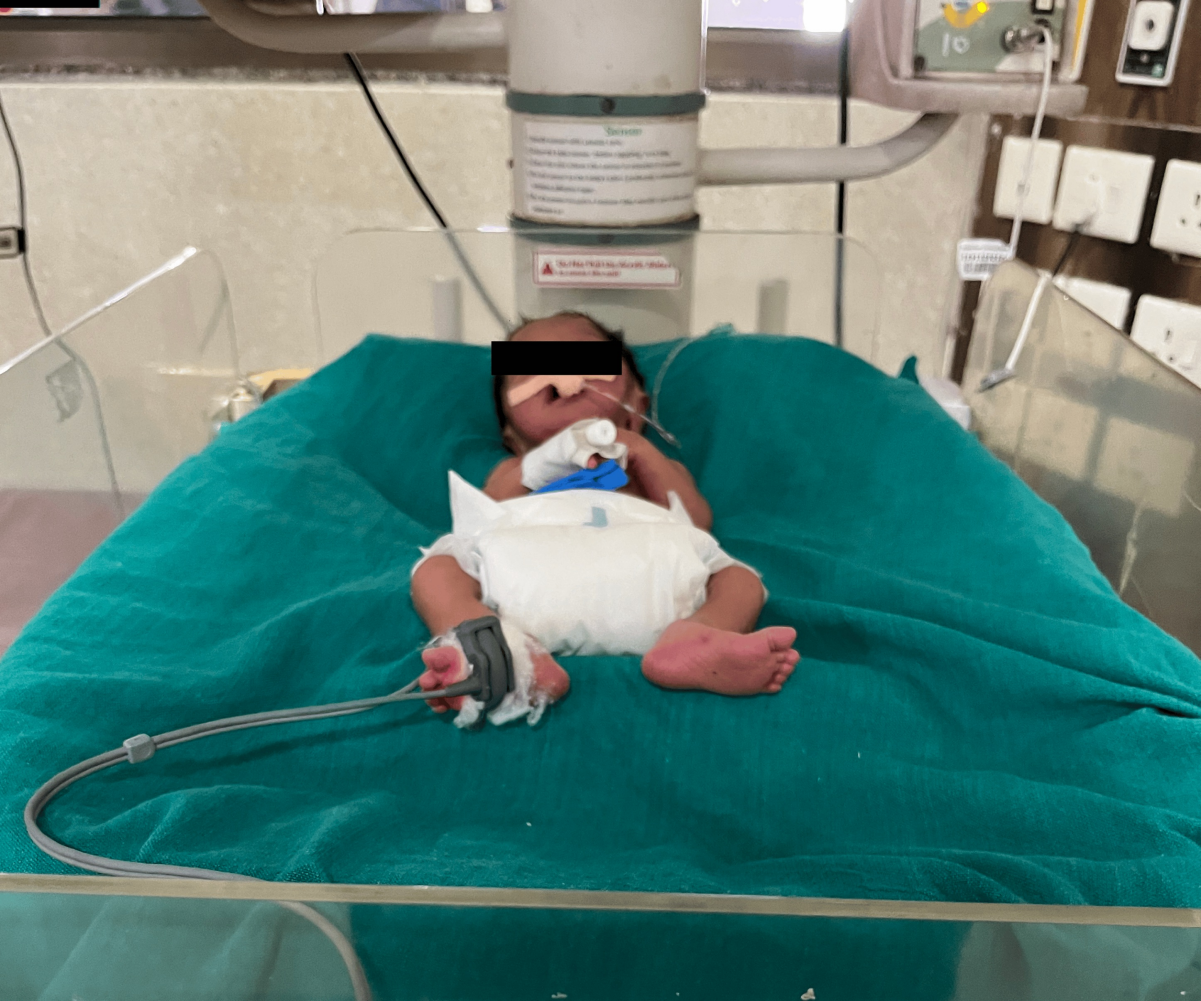
Positioning through the newly developed device

The first neonate was assigned to a group using a chit-based draw. Subsequently, participants were allocated alternately to the intervention and control groups in a sequential manner. Due to the alternating assignment procedure, the study followed a non-randomized controlled design, and allocation concealment was not applied. No blinding of physiotherapists or caregivers was feasible due to the visible nature of the positioning device. Participants in both groups received neonatal physiotherapy interventions, including visual stimulation, auditory stimulation, vestibular stimulation, tactile stimulation, joint range of motion (ROM), oro-motor stimulation, and sensory and self-calming activities, given once a day for approximately 20 minutes for six days a week, combined with positioning from the time of enrollment to completion of one week (seventh day). The interventions were planned according to the guidelines recommended by the American Physical Therapy Association [10,11]. Intervention group infants were positioned in the newly designed positioning device, and the control group was positioned in the conventional swaddling positioning in preterm neonates. At the end of each week, using valid tools, the postural development has been evaluated (Figure 1 and Figure 2).

Postural quality was assessed using the IPAT, a validated clinical instrument originally developed by Coughlin et al. [12]. The IPAT was used in its standard form, without any modifications, to evaluate outcomes in both the control and intervention groups, which is an open-access resource available in the public domain. IPAT scores were recorded from 0 to 10, with higher scores indicating more physiologically appropriate, flexed, and midline posture, and analyzed to determine the intervention’s effect on postural development. The IPAT demonstrates strong reliability (inter-rater intraclass correlation coefficient = 0.89, intra-rater ICC = 0.93) and established construct validity, supporting its use in evaluating postural development in the NICU.

Parents were included in treatment sessions and were encouraged to perform the recommended activities during their NICU visits. The intervention was carried out during the infant’s wakeful state, ideally before the next scheduled feeding. It was paused or stopped if the neonate showed signs of stress, such as fussiness, crying, or sleep. Throughout the intervention, neonates were closely monitored to promptly address minor concerns, including skin integrity issues, desaturation episodes, or signs of discomfort. These concerns were managed immediately by adjusting or halting the intervention, ensuring continuous safety and comfort.

None of the neonates exhibited any adverse signs. As part of the study, a novel postural support device was designed by the authors specifically for preterm neonates in the NICU. The newly designed postural support device was developed to replicate the flexed, contained intrauterine posture for preterm neonates. The device consists of a soft, medical-grade memory foam base with adjustable lateral supports, a head and neck stabilizer, shoulder rolls, and a lower-limb bolster. These components are interconnected using Velcro to allow individualized adjustment based on infant size and clinical needs. The device supports the head in midline, promotes gentle scapular protraction, and maintains the hips and knees in flexion with neutral alignment of the ankles and feet. Unlike conventional swaddling, which provides general containment but may allow hip abduction, shoulder retraction, or limb extension, the device provides structured and segment-specific support, helping to maintain physiological flexion, reduce postural asymmetry, and promote early neuromotor organization. For hygiene and comfort, a sterile cotton drape was placed over the device during each use. The device dimensions were based on ideal anthropometric data published by the Indian Academy of Pediatrics [13]. All components, including head and neck support, shoulder rolls, and knee bolsters, were interconnected using Velcro, allowing for easy adjustment to fit smaller infants. During use, the components were arranged on the device base and covered with a sterile hospital green drape to ensure hygiene and comfort. The device underwent thorough testing for safety, usability, hygiene, and ease of use. It demonstrated high clinical acceptability among NICU staff and caregivers, who found it easy to incorporate into routine care. A feasibility study was conducted to confirm its applicability [14].

**Figure 3:**
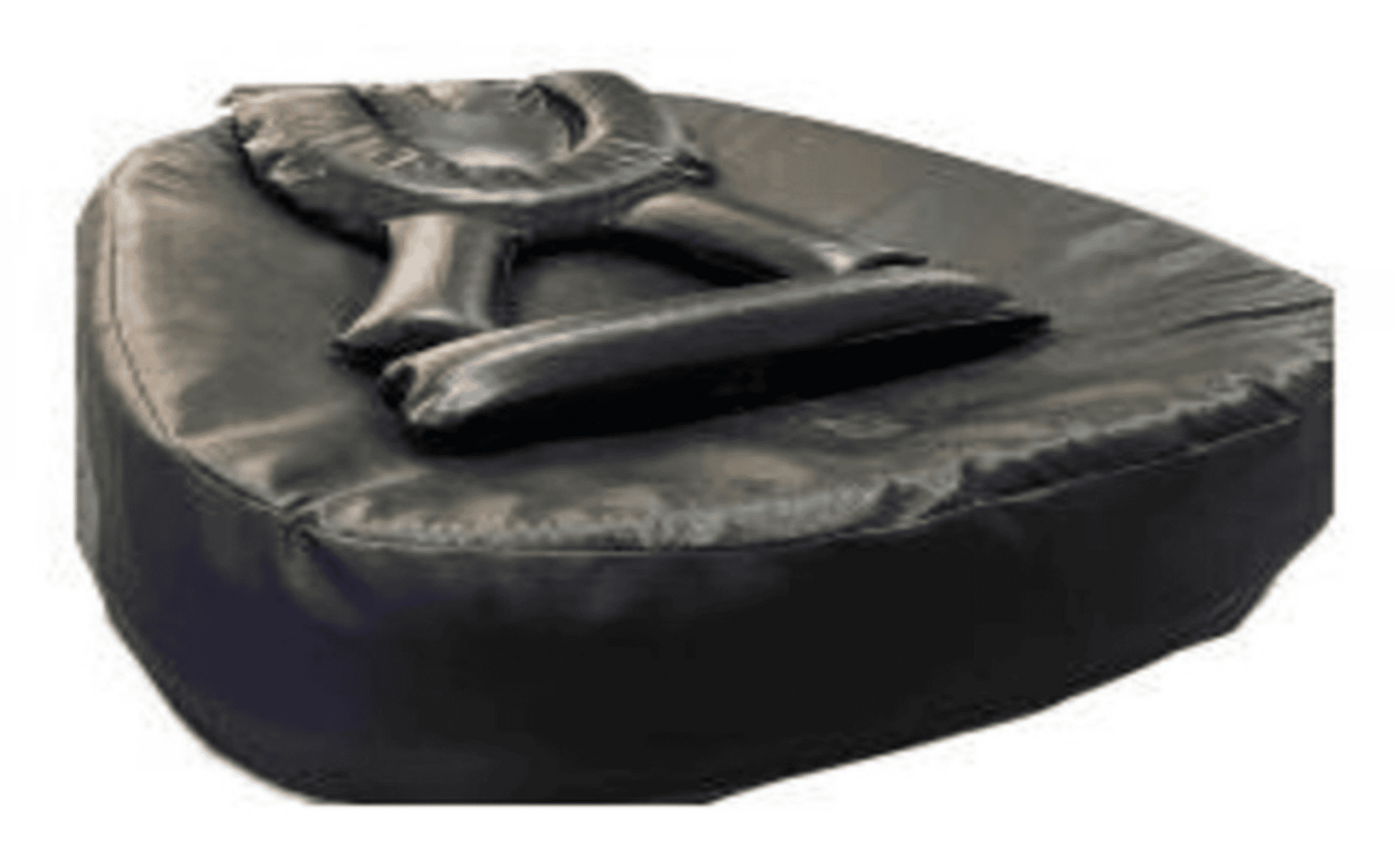
Postural supporting device Image credits: Dhwani D. Chanpura and Nalina Gupta

This “postural supporting device” is registered with design number 403807-001 (Figure 3).

Ethical approval

This study was approved by the institutional ethics committee (SVIEC/ON/PHYS/PhD/22012). After that, registration for the Clinical Trials Registry-India was completed, with registration number CTRI/2022/08/044708.

## Results

**Figure 4:**
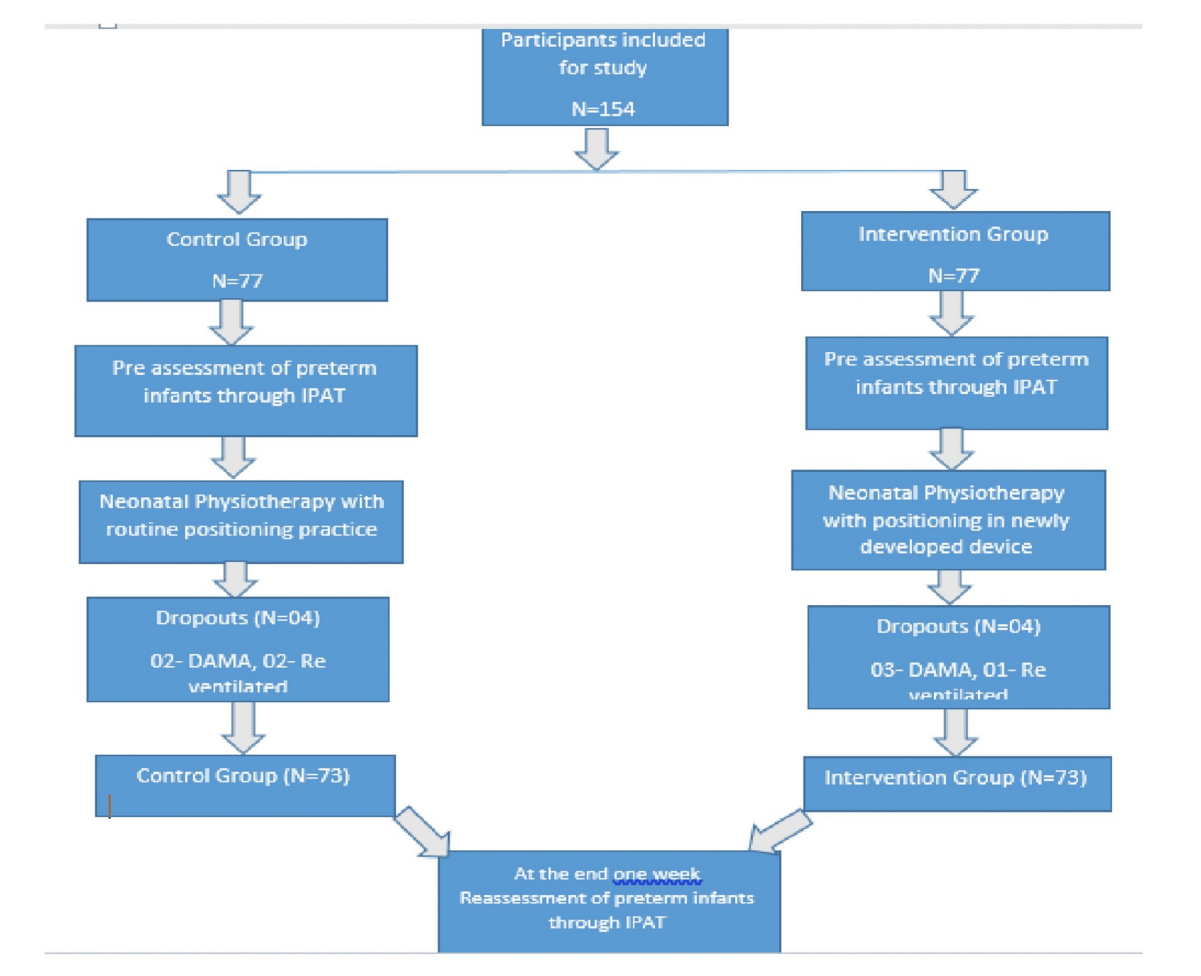
CONSORT flowchart CONSORT: Consolidated Standards of Reporting Trials, IPAT: Infant Positioning Assessment Tool, DAMA: discharge against medical advice

A sample size of 73 infants per group (total number = 146) was required to detect a mean difference of 40 with a standard deviation (SD) of 85 at 95% confidence and 80% power, calculated using the two-group comparison formula n = 2 × d2(Z1 ​+ Z2​)2 × SD2 (Figure 4). The study comprised 54.8% male neonates and 45.2% female neonates. Cesarean delivery was more common than vaginal delivery in both groups (63% versus 37% in controls, 53.4% versus 46.6% in intervention). Baseline comparisons showed no significant differences between groups, indicating comparability of groups at enrollment (Table 1).

**Table 1:** Comparison of the head and neck component of IPAT between the control group and the intervention group using an independent t-test IPAT adapted from Coughlin et al. [12] *Statistically significant IPAT: Infant Positioning Assessment Tool, SD: standard deviation

Component	Time point	Control (mean ± SD)	Intervention (mean ± SD)	Mean difference	t-value	p-value
Head	Pre	0.90 ± 0.29	0.73 ± 0.50	-0.178	-2.590	0.011*
	1 week	1.11 ± 0.31	1.05 ± 0.23	-0.055	-1.203	0.231
Neck	Pre	1.00 ± 0.00	0.99 ± 0.20	-0.014	-0.575	0.566
	1 week	1.11 ± 0.31	1.05 ± 0.23	-0.055	-1.203	0.231

At baseline, a significant difference was observed in head posture, with the control group showing higher mean scores compared to the intervention group (p = 0.011). However, by one week, no significant differences were noted between groups for either head or neck posture (p > 0.05), indicating comparable improvements in both groups.

As shown in Table 2, no significant difference was noted in shoulder posture at baseline (p = 0.411). However, by one week, the intervention group demonstrated significantly higher shoulder scores compared to the control group (p < 0.001), indicating marked improvement with the positioning device. For the hand component, baseline scores were significantly higher in the control group (p = 0.045). By one week, no significant differences were observed between the groups (p = 0.458), suggesting comparable progression in hand posture over time.

**Table 2:** Comparison of the shoulder and hand components of IPAT between the control group and the intervention group using an independent t-test IPAT adapted from Coughlin et al. [12] *Statistically significant IPAT: Infant Positioning Assessment Tool, SD: standard deviation

Component	Time point	Control (mean ± SD)	Intervention (mean ± SD)	Mean difference	t-value	p-value
Shoulder	Pre	0.79 ± 0.41	0.85 ± 0.40	0.055	0.824	0.411
	1 week	1.14 ± 0.35	1.52 ± 0.50	0.384	5.367	<0.001*
Hand	Pre	0.89 ± 0.46	0.74 ± 0.44	-0.151	-2.022	0.045*
	1 week	1.22 ± 0.45	1.16 ± 0.44	-0.055	-0.744	0.458

In Table 3, at baseline, no significant differences were observed between groups for hips/pelvis (p = 0.545) or knee/ankle/feet (p = 0.620). By one week, the intervention group showed significantly higher scores for both hips/pelvis (p < 0.001) and knee/ankle/feet (p < 0.001), indicating marked improvement in lower limb postural alignment with the positioning device.

**Table 3:** Comparison of the hips/pelvis and knee/ankle/feet component of IPAT between the control group and the intervention group using an independent t-test IPAT adapted from Coughlin et al. [12] *Statistically significant IPAT: Infant Positioning Assessment Tool, SD: standard deviation

Component	Time point	Control (mean ± SD)	Intervention (mean ± SD)	Mean difference	t-value	p-value
Hips/pelvis	Pre	0.90 ± 0.30	0.95 ± 0.50	0.041	0.607	0.545
	1 week	1.51 ± 0.50	1.97 ± 0.16	0.466	7.514	<0.001*
Knee/ankle/feet	Pre	0.97 ± 0.16	1.00 ± 0.44	0.027	0.497	0.620
	1 week	1.55 ± 0.50	1.93 ± 0.25	0.384	5.831	<0.001*

As presented in Table 4, no significant difference was found in the total IPAT score at baseline between the control and intervention groups (p = 0.402). By one week, the intervention group demonstrated significantly higher total scores compared to controls (p < 0.001), indicating greater overall improvement in postural development with the positioning device.

**Table 4:** Comparison of total IPAT between the control group and the intervention group using an independent t-test IPAT adapted from Coughlin et al. [12] *Statistically significant IPAT: Infant Positioning Assessment Tool, SD: standard deviation

Component	Time point	Control (mean ± SD)	Intervention (mean ± SD)	Mean difference	t-value	p-value
IPAT total	Pre	5.47 ± 1.24	5.25 ± 1.85	-0.219	-0.840	0.402
	1 week	7.63 ± 1.61	8.70 ± 0.88	1.068	4.976	<0.001*

Although small baseline differences were observed in certain components (head and hand posture), the pattern and magnitude of improvement across multiple postural domains consistently favored the intervention group, supporting the clinical relevance of the findings.

## Discussion

The present study investigated the impact of structured positioning combined with physiotherapy on postural development in preterm infants in the NICU, using the Infant Positioning Assessment Tool (IPAT). While both groups showed natural progress over time, the intervention group demonstrated more rapid and pronounced gains, with domain-specific improvements and significant advantages in several key postural areas.

In the head positioning domain, mixed results were observed across the study period. At baseline, the control group showed significantly better alignment (p = 0.011), possibly reflecting variability in natural positioning or early care practices. By week 1, both groups improved, and the difference was no longer significant (p = 0.231). The intervention group demonstrated steady progress, suggesting that structured physiotherapy and supportive positioning contributed to achieving midline head alignment, a crucial foundation for postural control and sensory motor development [15,16].

The gradual improvements in both groups align with natural developmental trends, as noted by the study, which reported that head control in preterm infants improves with age and environmental support [16]. Proper head positioning in early life is essential for preventing plagiocephaly, enhancing visual engagement, and supporting neurodevelopment [17,18]. Overall, the intervention appeared to correct early head positioning deficits, enabling comparable outcomes by week 1 and highlighting the value of consistent supportive positioning in facilitating midline control and symmetrical motor development.

In the neck positioning domain, no significant differences were observed between groups at any time point. Both groups began with nearly identical scores (p = 0.566) and showed gradual improvements over the first (p = 0.231), reflecting the natural course of neuromotor maturation in preterm infants.

These improvements are consistent with expected developmental trends, as infants gradually gain head and trunk control with NICU caregiving routines [16]. Although the intervention group did not show a statistically greater advantage, steady progress suggests that structured positioning and physiotherapy offered supportive benefits without overstimulation. The parallel improvements in both groups also align with findings highlighting the role of both standard NICU care and individualized interventions in promoting symmetrical neck control and healthy postural development [17].

Shoulder positioning, an important marker of postural symmetry and upper limb alignment, showed clear benefits in the intervention group. Baseline scores were comparable (p = 0.411), but by week 1, the intervention group demonstrated significantly better alignment (p < 0.001). These findings highlight the role of structured physiotherapy and supportive positioning in promoting shoulder flexion and midline orientation, key features of healthy postural development in preterm infants [17].

These findings align with previous research showing that interventions supporting flexion and containment improve shoulder posture and reduce asymmetry [17]. Poorly supported positioning in the NICU has been linked to scapular winging and instability, which structured developmental care can mitigate. The improvements seen in the intervention group likely reflect better neuromotor engagement and shoulder girdle tone, facilitated by therapeutic handling. Clinically, proper shoulder alignment is critical for self-regulation, reaching, and future gross motor skills, reinforcing the value of structured physiotherapy and positioning in optimizing postural development in preterm infants [16].

In the hands domain, the intervention group started with significantly lower scores than controls (p = 0.045), reflecting less optimal hand positioning. By week 1, both groups improved, and the difference was no longer significant (p = 0.458). The intervention group showed clear clinical gains, narrowing the baseline gap, suggesting that early physiotherapy and structured containment supported midline hand posture, a key milestone for self-regulation and early motor control.

Although differences were not significant beyond baseline, the early correction of asymmetry in the intervention group underscores its clinical value. Prior studies highlight that early support for hand-to-midline behavior promotes neurobehavioral organization and prepares infants for later fine motor skills [16,17,19]. In this study, the intervention helped correct early deficits, enabling comparable levels by week 1 and sustaining those gains, reinforcing the role of structured positioning in supporting early postural and motor development [19].

In the hips and pelvis domain, both groups started with comparable scores (p = 0.545). By the first week, the intervention group showed significantly higher scores (p < 0.001, mean difference = 0.466), indicating better hip flexion, alignment, and midline stability. These findings highlight the positive impact of structured positioning and physiotherapy on lower body postural alignment in preterm neonates [20].

Research shows that early pelvic flexion support improves trunk alignment, reduces extension posturing, and promotes healthy motor patterns [17,20,21]. The intervention group’s gains likely reflect stronger flexor tone and reduced asymmetry, which are crucial for preventing hip dysplasia and enabling milestones such as rolling, sitting, and crawling. Overall, the intervention had a significant and sustained effect on pelvic alignment, underscoring the value of structured positioning in supporting lower body posture and early neuromotor development.

In the knees, ankles, and feet domain, both groups began with similar scores (p = 0.620). By the first week, the intervention group demonstrated significantly better alignment and flexion (p < 0.001, mean difference = 0.384), highlighting the positive impact of structured positioning on lower limb posture and stability in preterm neonates.

The intervention promoted flexed, symmetrical lower limb postures, reducing positional deformities and supporting more natural movement patterns [21]. Such postural control is vital for neuromotor development and for achieving milestones such as kicking, rolling, and crawling. In summary, the intervention produced significant improvements in knee, ankle, and foot positioning, reinforcing the role of structured positioning in enhancing musculoskeletal organization and preparing preterm infants for functional motor development [16,17,20,21].

The total IPAT scores provided a comprehensive measure of overall postural quality and symmetry. At baseline, no significant difference was observed between groups (p = 0.402), confirming comparability. By the first week, the intervention group achieved significantly higher scores (p < 0.001, mean difference = 1.068), reflecting early and meaningful improvements across multiple domains, including head, shoulders, pelvis, and extremities. These findings align with prior research highlighting the role of structured, developmentally supportive positioning in promoting postural symmetry and midline orientation [16,17,20,22,23]. The consistent improvements in total IPAT scores suggest better integration of flexion, containment, and alignment, key to minimizing stress and fostering neuromuscular organization [20]. In summary, the intervention led to significantly greater overall postural gains, confirming the effectiveness of early and structured positioning strategies in preterm infants.

## Conclusions

This study suggests that the use of a novel postural support device, in combination with physiotherapy, may provide short-term benefits in promoting flexion, midline orientation, and overall postural alignment in preterm neonates during their NICU stay. While the intervention group demonstrated greater improvements in specific IPAT domains compared to conventional swaddling, these findings should be interpreted cautiously due to the short follow-up duration and the limitations associated with sample size. The results indicate a promising direction for structured developmental positioning; however, broader conclusions regarding long-term postural development outcomes cannot be made at this stage. Future research with larger samples and extended follow-up is recommended to validate these findings and determine whether early postural improvements translate into sustained developmental and functional benefits.
